# Co-Regulated Genes and Gene Clusters

**DOI:** 10.3390/genes12060907

**Published:** 2021-06-11

**Authors:** Sergey V. Razin, Elena S. Ioudinkova, Omar L. Kantidze, Olga V. Iarovaia

**Affiliations:** 1 Institute of Gene Biology Russian Academy of Sciences, 119334 Moscow, Russia; ioudinkova@inbox.ru (E.S.I.); o_kantidze@mail.ru (O.L.K.); iarovaia@inbox.ru (O.V.I.); 2 Faculty of Biology, M.V. Lomonosov Moscow State University, 119234 Moscow, Russia

**Keywords:** co-regulated genes, gene clusters, coordinated expression

## Abstract

There are many co-regulated genes in eukaryotic cells. The coordinated activation or repression of such genes occurs at specific stages of differentiation, or under the influence of external stimuli. As a rule, co-regulated genes are dispersed in the genome. However, there are also gene clusters, which contain paralogous genes that encode proteins with similar functions. In this aspect, they differ significantly from bacterial operons containing functionally linked genes that are not paralogs. In this review, we discuss the reasons for the existence of gene clusters in vertebrate cells and propose that clustering is necessary to ensure the possibility of selective activation of one of several similar genes.

## 1. Introduction

In living systems, there is often a need for a coordinated change in the expression level of a group of genes. A classic example is the activation of genes encoding the enzymes required for utilizing an alternative energy source in unicellular organisms. In prokaryotic cells, genes are grouped into operons, which are transcribed as polycistronic mRNAs [1,2,3]. In the genomes of the vast majority of eukaryotic organisms, classical operons (i.e., clusters of functionally linked genes that are transcribed as part of polycistronic mRNA) are absent. The only exception is the *Caenorhabditis elegans* genome, where a significant part of the genes is grouped into operons of various types. The most common operons are the SL1 type, the polycistronic mRNA of which is divided into a set of individual mRNAs through trans-splicing [4,5]. Single operons of this type have also been found in some other organisms [6]. At the same time, for unicellular eukaryotic organisms (yeast [7,8]), fungi [9,10]), and plants [11,12,13,14]), the organization of functionally linked genes into clusters is quite typical. Within the clusters, each gene has its own promoter, but there are also some regulatory elements that control all genes in the cluster. Thus, in the gene clusters encoding the synthesis of secondary metabolites in fungi, there is often a gene encoding a transcription factor that activates the promoters of the remaining genes of the cluster [15]. Being a trans-acting factor, for some reason, this activator only works within the gene cluster. The transfer of a target gene from a cluster to an ectopic position elsewhere in the genome, results in the loss of the ability to be activated by a cluster-specific regulatory factor. Along with cluster-specific (metabolic pathway-specific) transcription factors in fungi, there are also global regulators that coordinate the work of different metabolic pathways [15]. In other words, there is a multilevel regulatory system that is also common in mammalian and other vertebrate cells.

In fungi and plants, gene clusters often contain functionally related genes that are not paralogs. The reasons for the emergence and stabilization of such gene clusters in evolution are the subject of scientific discussions [16,17]. In vertebrate animals, gene clusters typically arise as a result of gene duplications, with subsequent specialization of the functions of proteins encoded by individual genes—the globin, histocompatibility complex, Hox, and olfactory receptor genes are examples of such paralogous gene clusters.

The coordinated activation of a large number of genes in eukaryotic cells is typically associated with the differentiation process, although there are exceptions, such as stress-response gene activation. Genes activated during differentiation can be either organized into clusters (e.g., clusters of globin genes) or scattered throughout the genome. In this review, we will analyze the mechanisms that control the expression of tissue-specific genes and examine the advantages of organizing such genes into clusters. The discussion will be largely based on the studies of vertebrate erythroid-specific genes. However, as necessary, we will refer to other tissue-specific genes and gene clusters.

## 2. Erythroid-Specific Genes

In the course of erythroid differentiation, transcription of a significant number of genes is activated, whereas many other genes are repressed. The final aim of erythroid differentiation is the generation of hemoglobin-producing cells. In vertebrates, hemoglobin comprises 4 polypeptide chains (two alpha- and two beta-type globin chains) and heme. In warm-blooded animals, genes encoding for the alpha and beta subunits of hemoglobin are segregated into two clusters, each containing several genes that are expressed at different developmental stages [18]. In fish and amphibians, the alpha- and beta-globin genes are located in a common cluster (or common clusters) [19,20,21]. However, these clusters also contain groups of genes that are specifically expressed in particular developmental stages. For many years, the mammalian and avian clusters of alpha- and beta-globin genes have served as the most popular models for studying the regulation of gene expression [22,23]. Each of these clusters is controlled by a super-enhancer, which in the beta- and alpha-globin gene clusters is referred to as the locus control region (LCR) [24] and major regulatory element (MRE), respectively [25]. In addition to the super-enhancer, a number of additional enhancers are involved in the control of globin gene expression. These enhancers can be located both within gene clusters and at a considerable distance from these clusters [26,27]. In globin-producing cells, all these enhancers are assembled into a single block, to which various globin genes are recruited in a stage-specific fashion [28,29,30,31].

The activity of enhancers is controlled by a set of erythroid-specific transcription factors (Gata1, Tal1, Klf1, and NFE2) [32]. The current model suggests that, being bound to an enhancer, these transcription factors recruit transcription machinery and trigger the assembly of a liquid activator compartment, through a physical process of liquid phase separation [33,34]. Transcriptional activation of individual globin genes correlates with the recruitment of their promoters to the activator compartment [30,31]. In accordance with this scenario, a number of studies have demonstrated that the same transcription factors are present both on the enhancer and on the promoters of genes controlled by this enhancer [35,36,37]. Due to the higher density of binding sites for transcription factors on enhancers, it is the enhancers that serve as the nucleation centers for the assembly of activator compartments [38,39,40]. The attraction of promoters to the activator compartment is controlled by architectural proteins. In erythroid cells, such architectural proteins are LMO2 and LDB1 [41].

There are significant differences in the transcription control mechanisms of the alpha- and beta-globin gene clusters [42]. A cluster of beta-globin genes is integrated into a more extended cluster of tissue-specific olfactory receptor genes. These genes are inactive in most cells. Accordingly, in the vast majority of non-erythroid cells, the extended genome region harboring both olfactory receptor genes and beta-globin genes is organized into inactive (DNase resistant) chromatin. In the course of erythroid differentiation, the chromatin status of the beta-globin gene cluster changes, as evidenced by an increase in the level of histone acetylation and the transition of the entire cluster to an active DNase-sensitive chromatin configuration [43,44]. This process is controlled by LCR and other enhancers located in the upstream region of the beta-globin gene cluster. The loss of this complex of enhancers as a result of the natural deletion of an extended genomic segment (Hispanic deletion) results in an inability to activate the beta-globin gene cluster in erythroid cells [45]. A cluster of alpha-globin genes is located in a permanently active region of the genome. It is surrounded by housekeeping genes and is found in an active (DNase-sensitive) chromatin configuration, in both erythroid and non-erythroid cells [42]. The main regulatory element of the alpha-globin gene cluster is located in the intron of an upstream housekeeping gene [46]. At least in the mouse, alpha-globin genes are transcribed in mixed transcription factories, along with adjacent housekeeping genes [47]. The current model postulates that the expression of both alpha- and beta-globin genes in erythroid cells is controlled by mechanisms operating at two levels [26,48,49]. The first level is the activation of the super-enhancer and the assembly of the enhancer block. In a cluster of beta-globin genes, this activation correlates with a change in the chromatin configuration of the entire gene cluster (a transition from DNase-resistant to DNase-sensitive configuration, which in turn, is ensured by an increase in the level of histone acetylation [44,45]). Super-enhancer activation is mediated by several erythroid-specific transcription factors, the most important of which are Gata1, Tal1, Klf1, and NFE2 [50,51]. The same factors activate the expression of other erythroid-specific genes that are not organized into clusters. The coordination of the expression of all erythroid-specific genes is provided primarily by the ability of their promoters to be activated by the above-mentioned erythroid-specific transcription factors. The expression of the transcription factors themselves is also coordinated in a certain way. According to a number of data, an important role in this process is played by the establishment of spatial contacts between genes that code for transcription factors. Architectural proteins, primarily CTCF [52], are involved in establishing such contacts. The binding of CTCF to recognition sites on DNA, in turn, can be modulated by specific non-coding RNAs [53]. Spatial contacts are also established between downstream targets of the key erythroid-specific transcription factors. Along with other factors, the recruitment of erythroid-specific genes to specialized transcription factories [54] contributes to the generation of an erythroid-specific gene interactome.

## 3. Functional Role of Gene Clustering in Vertebrate Cells

As aforementioned, the organization of functionally related genes into operons in prokaryotes and operon-like clusters in fungi and plants, is typically associated with the necessity to ensure coordination in the production of components of one protein complex or enzymes of one metabolic pathway. It is quite clear that such a function cannot be attributed to the above-discussed clusters of alpha- and beta-globin genes. Indeed, at each stage of development, preferably only one of the genes of each cluster is expressed. Even in cases when two globin genes of the same type are expressed (for example, in mice, βmajor and βminor adult globin genes), the production of one of them (βmajor) exceeds several times the production of the other. An important task is coordinating the expression level of alpha- and beta-globin chains. However, in warm-blooded animals, the corresponding genes are segregated in different clusters located on different chromosomes, and the coordination of their expression is provided by regulatory mechanisms of the first level. How this coordination is achieved is not entirely clear. Some observations suggest that in erythroblasts, the genes encoding the alpha and beta chains of hemoglobin are spatially close to each other [55]. However, this relative spatial proximity is far from a colocalization that may be expected for the genes attracted to a common transcription factory. At best, alpha- and beta-globin genes are often located close to the same speckle (SC35 domain), but the significance of this observation is not clear.

As globin gene clustering is not important for the hemoglobin subunit expression level coordination, what could the role of such clustering be? To answer this question, it is worth remembering that at each developmental stage, only one alpha-type globin gene and one beta-type globin gene are preferentially expressed. In mammals, there are beta-globin genes of the embryonic, fetal, and adult types and alpha-globin genes of the embryonic and adult types. Ensuring the expression of one of several similar genes is a rather difficult task that cannot be solved by regulatory systems of the first level. Apparently, this difficulty is due to the fact that the regulatory modules in the promoters of all globin genes are quite similar, and all these genes are, in principle, activated by the same transcription factors. We believe that precisely to make the choice of the gene that should work at a particular stage of development, second-level regulatory systems are required that work at the gene cluster level. The regulation is carried out in this case in cis, by creating alternative enhancer-promoter loops [30,31,56,57]. For this regulatory mechanism to work, it is important that the regulated genes are close to each other; that is, they are organized into a gene cluster. Within the cluster, the selection of genes to be expressed may be mediated by different mechanisms, including the ones that ensure selective repression of some genes of the cluster. The common theme is recruitment of co-repressors mediating histone deacetylation. In some cases, this is accompanied by DNA methylation [58,59,60,61,62].

How the spatial reconfiguration of globin gene clusters is directed is not entirely clear. There are many indications that an important role in this process is played by a change in the binding profile of CTCF, to recognition sites on DNA [52,63,64]. The interaction between CTCF binding sites is modulated by histone acetylation in the surrounding regions, which in turn is directed by erythroid-specific transcription factors, in particular GATA1 [65,66]. Non-coding RNAs can also influence the preference for CTCF binding to different recognition sites on DNA [53]. It is worth emphasizing once again that the correct switching of globin gene expression is possible only in the context of existing gene clusters. A simple change in the order of the genes, relative to the super-enhancer (for example, when the entire gene cluster is inverted), as well as the inclusion of an additional gene in the cluster, leads to serious disruptions in the switching process of both alpha- and beta-globin genes [67,68,69]. In experiments with ectopic expression, the adult beta-globin gene controlled by the LCR was expressed at an early stage of development, when this gene should not be expressed. The same gene in the construct, which additionally contained fetal-globin genes, showed correct stage-specific expression [70,71]. Apparently, the competition of different genes in the cluster for the LCR is essential for ensuring the stage-specific expression of individual genes.

The main conclusion that follows from the above discussion is that clustering of globin genes is necessary for the operation of mechanisms that ensure the selection of those genes from a number of paralogous genes that will be expressed in a particular situation. Similar trends can be traced in the case of other gene clusters of vertebrates. Hox gene clusters are a good example. In these clusters, the activation time of individual genes during development is determined by the order of the genes in the cluster [72,73]. As is the case for globin genes, Hox genes are subject to multilevel regulation through global regulatory elements located outside the gene clusters and additional enhancers [74,75,76]. Activation of various groups of Hox genes is associated with spatial reconfiguration of gene clusters; that is, it is provided by mechanisms that work in cis [77,78,79,80].

Odorant receptor genes are another example of clustered vertebrate genes. In humans and mice genomes, there are several clusters of odorant receptor genes located on different chromosomes. In the course of differentiation, transcription of only one of the odorant receptor genes is activated in an individual olfactory neuron. In the mouse genome, cluster-specific regulatory elements were found, which provided random activation of one of the genes in the cluster [81,82,83]. How coordination between different clusters is ensured, resulting in the expression of only the gene activated in one of the clusters, remains not entirely clear. According to a number of data, inactive genes from different chromosomes are combined in the repressive compartment [84,85]. From the standpoint of our discussion, however, it is important that in the case of olfactory receptor genes, clustering is required to select one of the genes with similar regulatory modules, which is determined by the cluster-specific regulatory elements [81,82,83,86]. A number of other examples of gene clusters can be cited, where out of several similar genes, only one is selected to be expressed. Thus, this process occurs in the human growth hormone (hGH) multigene cluster [87]. The same situation can be observed in clusters of protocadherin (*Pcdh*) genes. The mammalian genome contains three clusters of *Pcdh* genes (*Pcdhα*, *Pcdhβ*, and *Pcdhγ*). *Pcdhα* and *Pcdhγ* clusters are constructed in a similar manner—tandem arrays of genes encoding the first variable exon and then a group of three small constant exons. This genomic architecture is similar to that of immunoglobulin and the T cell receptor gene clusters. Within the *Pcdhα* and *Pcdhγ* clusters, each of the genes encoding the variable exon has its own promoter. The transcript of a variable exon initiated on this promoter is spliced to a common set of three downstream small constant exons, within the respective cluster. Hence, through stochastic activation of the promoter and cis-alternative splicing, clustered *Pcdhs* can generate dozens of different isoforms. The *Pcdhβ* gene cluster contains only variable genes, each of which encodes a full-length protein. The *Pcdhα* cluster is regulated by a 3′super-enhancer consisting of two cis-regulatory elements. The expression of *Pcdhβ* and *Pcdhγ* clusters is controlled by a super-enhancer located in the 3′-region of the *Pcdhγ* cluster [88]. The assembly of enhancer-promoter loops is mediated by CTCF, the binding sites of which are present in all promoters and enhancers, and in the *Pcdhα* cluster, the promoter of each alternative gene is flanked by two CTCF binding sites (an additional one is located in the coding region). Enhancer-promoter loops are formed between forward and reverse CTCF-binding sites via CTCF/cohesin-mediated chromatin loop extrusion. Chromatin loop formation is regulated epigenetically through the methylation of CpG dinucleotides at the CTCF binding sites on the *Pcdhs’* promoters [89]. Accordingly, demethylation of the CTCF-binding site at a particular promoter allows the interaction of this promoter with the enhancer, resulting in activation of expression of this particular *Pcdhα* gene [90]. Recent studies have shown that the aforementioned additional CTCF site of each alternative exon is associated with an antisense promoter that controls the transcription of long non-coding RNA (lncRNA). Stochastic transcription of this RNA through the sense promoter, triggers DNA demethylation of the corresponding CTCF-binding site, allowing CTCF binding and subsequent activation of the sense promoter [90]. The selection of the Pcdh promoter via the establishment of specific patterns of CTCF binding sites’ methylation across the gene cluster, occurs early in the course of embryonic stem cell differentiation, and is then stably inherited by differentiated neurons [91]. Bioinformatic analysis also revealed several other gene clusters, organized similarly to *Pcdh* gene clusters [92]. Clearly, in all these gene clusters, the clustering of similar genes is necessary to select one from many genes, which will be expressed in a particular cell. In globin and *Pcdh* gene clusters, this selection is ensured by establishing alternative 3D cluster configurations. However, other mechanisms are also possible. Thus, immunoglobulin gene clusters are rearranged at the DNA level [93].

Regarding tissue-specific genes not being organized into clusters, their expression can be controlled both by the regulatory systems of the first level (direct activation of promoters by tissue-specific transcription factors) and in two stages—activation of a tissue-specific enhancer and subsequent activation of secondary targets. The genome distribution of genes not organized in dense clusters is not entirely random [94,95,96]. Several observations suggest that they can form sparse clusters corresponding to the structural and functional blocks of the genome (such as TADs or insulated neighborhoods), which limit the areas of tissue-specific enhancer activity [97,98,99]. However, genome-wide analysis did not reveal a good match between TADs and the co-expression domains [100].

## 4. Gene Cluster Evolution

**Figure 1:**
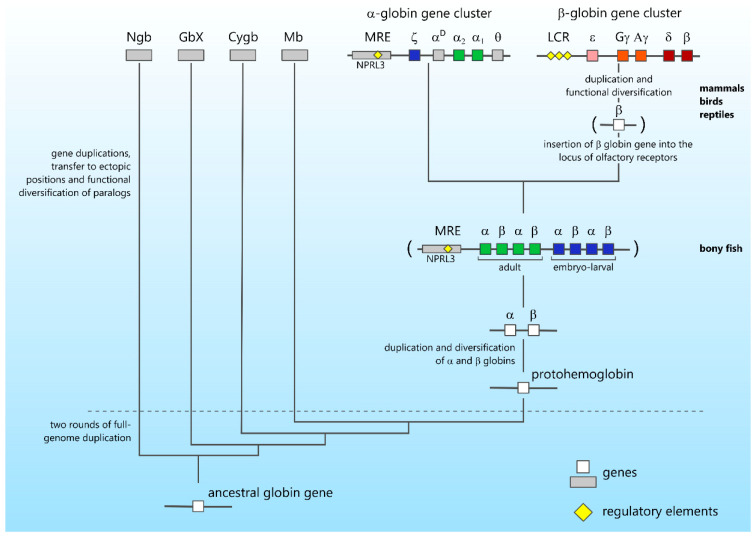
Main globin gene evolution stages. Clusters of human alpha- and beta-globin genes are shown at the top of the evolution tree. In the alpha-globin gene cluster, the colors of embryonal and adult globin genes show that these genes are homologs of embryonal and adult globin genes present in the ancestral cluster. The different color scheme for designation of embryonal (ε), fetal (Gγ, Aγ), and adult (δ, β) globin genes in the beta-globin gene cluster is used to show that these genes are not homologs of embryonal and adult globin genes present in the ancestral cluster, but originated via duplication of a single beta-type globin gene transposed to a new genomic position.

The evolution of gene clusters can be traced by the example of clustered globin genes (Figure 1). Several interesting reviews have been devoted to the role of duplications in globin gene evolution [101,102]. The ancestor of globin genes is believed to simultaneously appear in the eukaryotic genome with mitochondria and plastids, as a result of gene transfer or genome fusion [103]. Most likely, the functions of the ancient membrane-bound globin were related to signaling or protection [104]. The family of globin genes arose as a result of neo- and sub-functionalization of additional copies of genes that arose after whole-genome and segmental duplications, which repeatedly occurred during the evolutionary process [105,106,107]. In the genomes of modern vertebrates, there are eight groups of globin genes—neuroglobin (*Ngb*), cytoglobin (*Cygb*), androglobin (*Adgb*), hemoglobin (*Hb*), and myoglobin (*Mb*), which are present in almost all vertebrate genomes, as well as the less common globins X, Y, and E (*GbX*, *GbY*, and *GbE*) [106,107]. Protoneuroglobin is believed to be the common ancestor of the entire family of globin proteins in both protostomes and deuterostomes [108]. Gene duplications have played an exceptional role in vertebrate evolution. The two rounds of whole-genome duplications (WGDs) that occurred at the dawn of vertebrate’s radiation provided fuel for the evolutionary process, and made possible the emergence of fundamentally new traits and regulatory systems [109,110,111,112,113,114,115,116]. As a result of two WGD cycles, specialized globins that enabled the storage (Mb and Cygb) and transport (Hb) of oxygen appeared in the common ancestor of vertebrates. The specialization and functional diversification of globin genes was related to both genome-wide and tandem duplications, which repeatedly occurred with globin genes [117,118]. Tandem duplication, which resulted in the formation of alpha- and beta-globin genes, occurred 450–500 million years ago in the Ordovician, before the separation of cartilaginous fish and the common ancestor of modern tetrapods [117,119]. It is assumed that the genomic context and structure of the postduplication ancestral locus was similar to the structure of the major locus of alpha-/beta-globin genes of modern teleost fishes [20]. Within the locus, alpha- and beta-globin genes alternate and are transcribed in opposite directions. The organization of alpha- and beta-globin genes into a common cluster controlled by an upstream regulatory element that is typical for fish is also preserved in amphibians [120,121,122,123]. It should be noted that the emergence of clustered globin genes in the course of evolution correlates with the diversification of these genes in terms of expression at particular developmental stages. For example, in the *Danio rerio* genome, the major locus of globin genes is structurally and functionally segregated into two stage-specific subdomains, which contain genes that are expressed during the adult and embryonic-larval stages of development [19,124].

The next important stage in the evolution of globin genes was the segregation of alpha- and beta-globin genes in amniotes. The key event in this case was the relocation of a part of the ancestral cluster or, more likely, one gene pair to a new genomic position (into the odorant receptor gene cluster) [20,120,125,126]. The subsequent loss of the alpha-globin gene(s) and duplications of the ancestral beta-globin gene gave rise to modern clusters of beta-globin genes. It should be mentioned that the specialization of embryonic and adult genes within the alpha-globin gene cluster occurred within the ancestral domain [127], whereas the emergence of stage-specific forms in the domain of beta-globin genes occurred as a result of a set of duplications after the transfer of the ancestral beta-globin gene to another chromosome [128] (see schematic in Figure 1). Duplications of beta-globin genes occurred independently in mammals and birds (i.e., stage-specific forms of hemoglobin were invented several times during evolution) [128,129]. It should be mentioned that gene duplications occur relatively often. However, in most cases, one of the duplicated copies is lost in the course of subsequent evolution (turns into a pseudogene). For example, the platypus alpha-globin gene cluster contains the non-functional beta-type omega globin gene [125,130]. The diversification of globin functions into embryonic, fetal (primate-specific), and adult ones contributed to the preservation of duplicated globin genes, which resulted in the emergence of the gene cluster [18,128].

As already mentioned, gene cluster integrity is important for the normal expression of the genes contained in the cluster. Moving one or more genes of the cluster to an ectopic position excludes these genes from the regulatory chain. However, such genes retain tissue-specific promoters, which, under certain circumstances, can be integrated into another regulatory network. As aforementioned, gene duplicates are often lost in the course of evolution. However, moving them to an ectopic genomic position may contribute to their preservation and neofunctionalization, in accordance with the new genomic context [131]. Apparently, this process contributed to the diversification of the functions of neuroglobin and other globins, as well as other non-clustered globin genes. In this regard, it is also interesting to trace what happens to foreign genes, which were moved to the locus of tissue-specific genes, due to genomic rearrangement. A cluster of tissue-specific genes is a single regulatory domain under the control of the first-level regulatory systems that direct tissue-specific expression. An alien gene trapped in this regulatory network most likely will not be able to perform its original function. However, there is a possibility that the promoter of this gene will eventually be adapted to work in the regulatory context of the gene cluster. An example of such a scenario is the chicken *TMEM8* gene, which was relocated downstream of the alpha-globin cluster, as a result of chromosomal inversion. Being relocated to the domain of chicken alpha-globin genes, this gene encoding a transmembrane protein was included in the regulatory network of this domain and acquired an erythroid-specific expression profile [132]. A similar scenario can be traced in the case of the folate receptor gene, which in the chicken genome, is located close to the cluster of beta-globin genes. Although this gene is regulated independently of the genes of the beta-globin cluster, it nevertheless acquired an erythroid-specific expression profile [133].

## 5. Concluding Remarks

**Figure 2:**
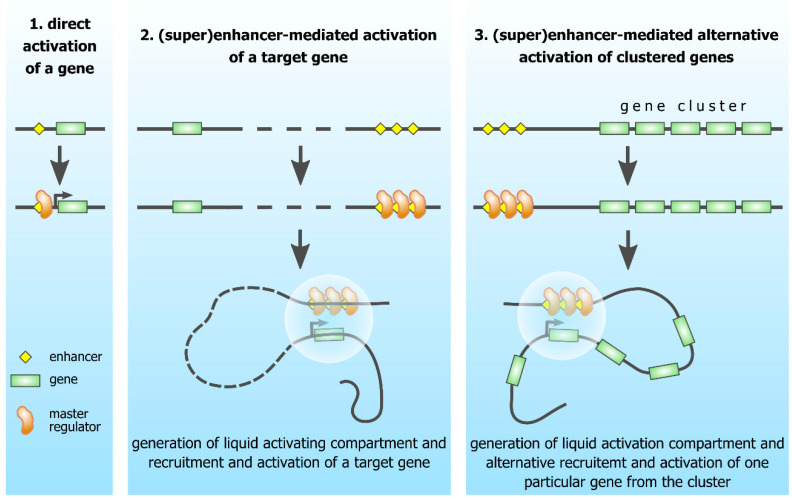
Strategies of transcriptional control of dispersed and clustered tissue-specific genes.

In sum, we can state that in the genomes of the vast majority of eukaryotes, the coordinated expression of a number of genes (for example, genes encoding enzymes of one metabolic pathway, genes for which the expression begins in response to hormone action, or tissue-specific genes in the course of differentiation) is not conjugated with the organization of these genes into functional clusters. This coordination is provided by specific transcription factors that either directly interact with the promoters of the activated genes or activate a specific set of enhancers, to which, in turn, the genes dependent on these enhancers are recruited (Figure 2). The loss of operons in the genomes of most eukaryotic organisms is apparently associated with a significant increase in the number of co-regulated genes, and the need to activate different combinations of genes in response to specific stimuli. In this situation, clusters of functionally linked genes are useless. At the same time, clustering turned out to be in demand for solving a different biological problem. In eukaryotic genomes, gene clusters typically contain paralogous genes resulting from tandem duplications. In the course of evolution, there has been a certain functional diversification of paralogous genes associated with their adaptation, to perform similar but still different functions. Accordingly, in specific situations, it becomes necessary to activate the expression of only one or several similar genes. It is this task that is solved at the level of gene clusters, via spatial (and sometimes genomic) reconfiguration of the gene cluster, which ensures the selective attraction of one of the genes to the enhancer (Figure 2).

## Data Availability

Not applicable.
